# Authors' reply re: SSRI use during pregnancy and risk for postpartum hemorrhage: a national register‐based cohort study in Sweden

**DOI:** 10.1111/1471-0528.16585

**Published:** 2020-11-23

**Authors:** A Skalkidou, I Sundström Poromaa, E Elenis

**Affiliations:** ^1^ Department of Women’s and Children’s Health Uppsala University Uppsala Sweden

*Sir,*

We thank Dr Sholapurkar for showing interest in our register‐based study on the risk of postpartum haemorrhage (PPH) in relation to depression and use of selective serotonin reuptake inhibitors (SSRIs) and for opening an important debate.^1^

In his letter, Dr Sholapurkar raises possible limitations with register‐based data. Specifically, that with large sample sizes, small effect sizes can be identified as statistically significant. What is a clinically relevant effect size is still a topic for debate. We would argue that an odds ratio of about 1.3 for having a postpartum bleeding of more than 1000 ml (a strictly pre‐defined outcome in our study)^2^ is not minimal. When dealing with prevalent exposures such as depression with/without use of SSRIs (at 20%), the public health impact of relatively small effect sizes is considerable.

Also, women who have suffered from depression are at higher risk for postpartum depression (PPD). PPH, and especially postpartum anaemia and a negative delivery experience (often following a PPH), are further risk factors for PPD^3^. PPD has a devastating effect on women and the whole family and costs about US$ 32,000 per mother.^4^ In this context, we believe that further evaluation of extra vigilance and consideration of practical preventive measures, with low costs and associated risks, should be further assessed. Preventing even some PPH cases among these high‐risk groups not only would alleviate suffering but also seems like a highly cost‐effective measure.

It was argued in the letter that in clinical situations, one would not need to take any extra precautions for a patient with 9% risk for PPH instead of 7% and that other risk factors such as twin pregnancies are far more important. We of course agree that twin pregnancy confers much higher risk, and that is why we have specifically excluded these from our study, to focus on a low‐risk population. A possible interaction effect of SSRI use and twin pregnancy should be further tested. Blood loss as a continuous variable was also analysed, confirming the association of SSRIs with increased postpartum bleeding, but it was not included in the article because of word constraints. We believe that the chosen primary outcome is much more clinically relevant.

In research in general, and with large datasets specifically, there is a risk of selective reporting of specific analyses, as indirectly implied by the exposure‐wide study by Patel et al.^5^ cited by Dr Sholapurkar. This was nevertheless not the case for this study, which was founded on a hypothesis based on solid pathophysiological grounds; SSRIs increase bleeding tendency in non‐pregnant populations. In the case of a potential association between SSRIs and PPH, as Dr Sholapurkar writes, there have been conflicting results. Indeed, real‐world observational data do not allow for full control of confounders, and every dataset may have its own biases. As the question would hardly be ethical to investigate in a randomised controlled trial, we strove to advance knowledge by reproducing previous results in a real‐world Swedish setting. However, we acknowledge that further studies, preferably with a complete preregistered study protocol, are needed.
